# Bacteriophages in the Rhizosphere: Roles in Nutrient Cycling, Bacterial Community Structure, and Animal‐Mediated Dispersal

**DOI:** 10.1002/mbo3.70330

**Published:** 2026-06-23

**Authors:** Majid Komijani, Hassan Maddahi, Marzieh Rezaei, Mohammad Hussein Abnosi, Abdullah Khalaf Ahmed

**Affiliations:** ^1^ Department of Biology, Faculty of Science Arak University Arak Iran; ^2^ Department of Cell & Molecular Biology and Microbiology, Faculty of Science and Biotechnology University of Isfahan Isfahan Islamic Republic of Iran

**Keywords:** animals, bacteriophage, dispersal mechanisms, elemental cycles, plant health, rhizosphere, soil

## Abstract

The rhizosphere, a critical soil layer around plant roots, is enriched with carbon from root exudates, influencing microbial communities that can either protect against or cause plant diseases. Bacteriophages significantly impact soil nutrient cycles and ecosystem processes through cell lysis and horizontal gene transfer. They play a vital role in the rhizosphere by affecting plant stress responses and climate adaptation. Bacteriophages exert a range of negative effects on *Actinobacteria*, impacting their ecological and physiological functions by diminishing *Actinobacteria'*s roles in antibiotic production, soil health, and plant growth. Phage predation affects nutrient cycling by influencing nitrogen and carbon metabolism, with evidence showing that phages can alter microbial diversity and function, leading to changes in soil ammonium levels and carbon decomposition rates. In wastewater treatment, bacteriophages can improve process efficiency by targeting harmful bacteria, managing foam formation, and enhancing sludge reduction through enzymatic action. Additionally, bacteriophage dispersal mechanisms in the rhizosphere can be enhanced by rhizosphere‐associated animals. Numerous invertebrate and vertebrate animals can significantly alter the rhizosphere environment by amplifying, mobilizing, and distributing both phages and bacterial hosts. Herein, three main mechanisms by which animals enhance the dispersal of bacteriophages in the rhizosphere are discussed. This review discusses bacteriophages' roles in soil ecosystems, highlighting their impact on nutrient cycling, plant health, and soil remediation, as well as animal‐mediated phage dispersal mechanisms. Overall, while bacteriophages have potential biotechnological applications, their negative effects on microbial functions and nutrient cycling highlight the need for balanced use and further research.

## Graphical abstract

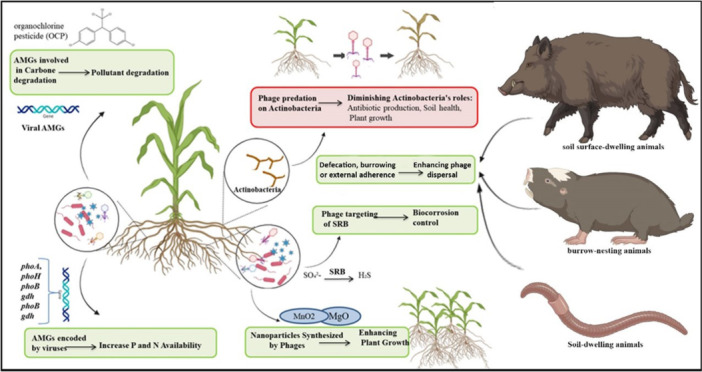

## 1 Introduction

The region of soil 2–5 mm from the roots, called the rhizosphere, is clearly distinct from the surrounding bulk soil. There, a large pulse of C molecules is flowing from the roots, which release photosynthetically assimilated C through the rhizodeposition process (Philippot et al. 2013). Microbes in the soil can either promote disease or provide some protection (Xu et al. 2020; Wang et al. 2021). The species and activities of these same microbes show marked spatial variability (Shi et al. 2018).

Bacteriophages have been shown to play important roles in nutrient cycling, food webs, and genetic diversity through cell lysis and horizontal gene transfer (Jardillier et al. 2005; Suttle 2007).

Key functions in the rhizosphere are assumed for these unknown genes, whereby they might influence abiotic stresses in plants and therefore impact plant resistance as well as adaptation to climate change (Pratama et al. 2020).

They maintain the turnover of bacterial mass through recurrent infections (Daly et al. 2019) an and they have an impact on ecosystem processes like the functioning of the soil nitrogen cycle (Braga et al. 2020), carbon cycling (Albright et al. 2022) or pollutant degradation, representing a new route for using viruses to clean polluted soils in search of remediation strategies (Zheng et al. 2022).

The most abundant type of phage in soil systems remains to be characterized. Tailed phages, such as members of the families Myoviridae, Podoviridae, and Siphoviridae, are thought to be the most common forms in the soil virome, but their diversity is also undoubtedly underestimated (Pratama et al. 2020).

These phages influence community structure and regulate the soil microbiome by altering ecological functions (Suttle 2007), They control host bacterial populations and diversity through cell lysis (Brum and Sullivan 2015), reprogram bacterial energy metabolism by triggering the expression of virus‐encoded auxiliary metabolic genes (AMGs) (Roux et al. 2016).

These genes are dispensable for phage propagation and replication but affect host metabolism (Pratama et al. 2020). Soil viruses support bacterial host to cope with various environmental challenges omics encyclopedia of genes associated with AMG in soil virome such as energy metabolism (Zheng et al. 2022), stress response and xenobiotics degradation under pesticide contamination in soil (Chao et al. 2023), detoxification of metal to achieve evolutionary adaptation for bacterial community against arsenic insult (Tang et al. 2023), sulfur metabolism (Kieft et al. 2021), Phosphorus cycle (Gao et al. 2022), nitrogen cycle (Gazitúa et al. 2021), and organic carbon cycling to help break down complex organic matters by the host (Zhao et al. 2022).

The present review describes the most recent knowledge on the impact of bacteriophages as key players in environmental performance within the soil habitat. Our analysis starts by reviewing the potential beneficial and detrimental effects of bacteriophages on the surrounding environment, via predation on host bacteria. The subsequent section provides an overview of the animal taxa and mechanisms for phage movement in the rhizosphere.

## 2 Eco‐Evolutionary Role of Bacteriophages as Predator by Preying Host Bacteria

Being predators of host bacteria, phages may influence environmental cycles. When these bacteria are participating in nutrient cycling (e.g., carbon, nitrogen, phosphorus, sulfur), the bacteriophage‐induced lysis may affect their pool size, community structure, function, and metabolism. This has implications for nutrient turnover, availability, agricultural output, and Greenhouse gas emissions (Wu et al. 2025).

The ecological impact of phages, either beneficial or detrimental, is associated with the role their hosts perform. Should the bacterial hosts be associated with pollutant degradation, phage therapy may reduce the feasibility of bioremediation (Ye et al. 2019).

In contrast, in the case of pathogenic host bacteria carrying ARGs, phage activity might be advantageous for ecosystem health. The effect of phage therapy on ARG transmission is affected by different environmental factors, including soil physicochemical properties, indigenous microbiota, and climatic conditions. These all together should influence the dynamics of ARG transmission (Ye et al. 2019).

Even though phage therapy is presumed to have a minimal effect on ARGs distribution because of the low transduction frequency in the environment (Calero‐Cáceres and Balcázar 2019; Lekunberri et al. 2017), and sparsity of ARGs in phages (Enault et al. 2017), some studies have reported that ARB and ARG abundances decline in soil after addition of phages. This suggests that the released ARGs from lysed host bacteria are further decomposed (Calero‐Cáceres and Balcázar 2019; Lekunberri et al. 2017).

## 3 Beneficial Impact of Bacteriophages on Soil Environments

### 3.1 Enhancing Crop Yield by Reducing Phytopathogen Virulence in Soil

It has been demonstrated in many case studies that soil associated with plant roots is a unique physical, biochemical, and ecological boundary. This region is largely influenced by the root system, which exudes a variety of chemicals into the soil (Nardi et al. 2000; Bais et al. 2001).

Some of the phages are found in 6 out of 10 most important bacterial phytopathogens, such as *Pseudomonas*, *Ralstonia*, *Xanthomonas*, and *Xylella* (Mansfield et al. 2012). They are also present in *Dickeya*, *Erwinia*, and *Pectobacterium*‐related bacteria. And further, phages from genera such as *Acinetobacter, Clostridium*, and *Pseudoalteromonas* are also significant as they affect the plant‐forming part of the remediation process. These findings are important because the phages of *Propionibacterium* and *Yersinia*, both opportunistic human pathogens, can colonize plants for reasons yet undetermined and must be controlled (Sharma et al. 2019).

Phages can promote plant health and growth by favorably affecting the tripartite interaction of phage, bacteria, and plant (Sharma et al. 2019). An instance of such a tripartite relationship is the regulation of *Xanthomonas campestris* pv. *campestris* (Xcc) to plants after phage application. *Xcc* is a crucifer‐pathogenic bacterium that causes black rot disease in cruciferous vegetables such as broccoli, cabbage, cauliflower, and radish, which causes significant yield loss worldwide (Qian et al. 2005).

In plants, Phage Xccφ1 acts against infection of Xcc by degrading its bacterial biofilm architecture. Papaianni et al. were able to disrupt several metabolic pathways of amino acids, lactate, and galactomannan that are crucial for the maintenance of biofilm integrity in combination with Xccφ1 supplemented with either mineral hydroxyapatite or eicosanoic acid (C20:0) (Papaianni et al. 2020).

Bacterial biofilm formation is dependent on amino acid metabolism (Wong et al. 2015). The glutamate increased, while arginine, glycine, 3‐methylhistidine, isoleucine, leucine, lysine, methionine, phenylalanine, pyroglutamate, tyrosine, and valine levels were reduced after treatment with phage Xccφ1 (Papaianni et al. 2020). Enhanced formation of biofilms (Goodwine et al. 2019) was mediated by bacterium adhesion utilization (Ene et al. 2012), and not a result of high levels of lactate, as is understood from the results here, which show low levels in treated groups, meaning that lytic action for Xccφ1 needs to take place under lower concentrations (Lee et al. 2014). Phages also produce saturated fatty acids in bacterial membranes, which could indicate cell lysis. Furthermore, increased galactomannan promotes *X. campestris* biofilm viscosity and contributes to biofilm degradation (Flemming and Wingender 2001).

This approach could be applied to the study of other biofilm‐forming bacteria, for example, *Escherichia coli* and *Pseudomonas aeruginosa*, where Pf4 phage has been demonstrated to depress the metabolic activity of *Aspergillus fumigatus* biofilms (Penner et al. 2016).

The phage‐induced inhibition was dose‐dependent and abrogated by denaturation of the phages, being more effective against preformed *A. fumigatus* biofilms than on biofilm formation. Pf4 has a strong affinity for *A. fumigatus* hyphae, and at the thickness of the biofilm, it binds iron wherefore starving of this precious element. Ferric iron can reverse Pf4‐mediated inhibition, and preexisting biofilms are more resistant (Penner et al. 2016).

*P. aeruginosa*, a common soil resident, has been reported to cause severe infections in plants and serves as a potential plant pathogen (Rahme et al. 1995; Silo‐Suh et al. 2002). Tarakanov et al. controlled that of *Pseudomonas savastanoi* pv. *glycinea* (*Psg*) in soybean through bacteriophages (Tarakanov et al. 2022).

They evaluated the efficacy of single phages against several *Pseudomonas* spp. and other phytopathogenic bacteria. A major part of the phages was able to infect Psg strains. Phage therapy provided a reduction in bacterial blight development in soybean by 50% in comparison with the control. They found that phages are promising biocontrol agents for various strains related to *P. savastanoi*. In addition, there were avirulent *Bradyrhizobium japonicum* from soybean seed treatment and their safety to traditional nitrogen fixers (Tarakanov et al. 2022).

Another research investigated imposing the virulence of phytopathogens via infection with filamentous phages. Filamentous phages are a distinct group of single‐stranded DNA viruses characterized by an elongated spiral protein shell, no lipid membrane, and a small, compact genome. Unlike lytic phages that kill their hosts, filamentous phages establish chronic infections without host lysis, continuously extruding new virions while the bacterium survives and replicates. These phages increase the colonization efficiency and genetic stability of bacterial inoculants, enabling them to outcompete native bacteria in the rhizosphere and reduce phytopathogen abundance. This concept has been validated across multiple studies: when combined with plant growth‐promoting rhizobacteria (PGPR), filamentous phages contribute to direct effects (root elongation, improved soil nutrition, enhanced nutrient uptake) and indirect effects (pathogen suppression, contaminant reduction) that support land reclamation and ecosystem restoration (Rakonjac 2022).

It indicated that phages were a promising agent against bacterial wilt on crops by reducing the pathogen's virulence. Addy et al. (2012) reported multiple biological and physiological changes, such as the loss of virulence in *Ralstonia solanacearum*, the causal organism of bacterial wilt, following infection with φRSM phages (Addy et al. 2012).

The down‐regulation of *phcA* and *phcB* (global virulence regulator [Addy et al. 2012]) in φRSM3‐infected cells indicated an inadequate range of activation of the virulence genes. Tomato plants presented no wilt symptoms when inoculated with φRSM3‐infected *R. solanacearum* strains. First, disruption of the *orf15* gene encoded by φRSM3 resulted in restoration to wild‐type virulence levels and *phcA* expression levels (Addy et al. 2012), thereby indicating that orf15 functions as a repressor of *phcA* and is itself involved in attenuation.

Despite the fact that many studies have reported on the successful use of phage as an effective alternative to conventional approaches for the control of bacterial diseases in plants (Gill et al. 2003; Obradovic et al. 2005; Jackson and Jones 2004; Jones et al. 2006, 2007; Balogh et al. 2008; Obradovic et al. 2008; Fujiwara et al. 2011; Gašić et al. 2011; Murugaiyan et al. 2011), treatment with bacteriophages is not considered a suitable strategy for controlling plant pathogenic bacteria, due to the inconsistency, and narrow host spectrum associated with their activity (Summers 2005).

### 3.2 Phage‐Enhanced Bacterial Inoculants Boost Plant Health and Growth

Phages to provoke modifications influencing plant development, whether directly or indirectly. Sharma et al. (2019) in a meta‐analysis, and as a future perspective, some research directions were suggested in investigating the phage–bacteria–plant interaction. Filamentous phages increase the advantages of bacterial inoculants, since they increase the degree of colonization and stability in their genotypic contents to reduce phytopathogen abundance and contaminant levels during complementation with filamentous‐phage‐free inoculants (Sharma et al. 2019).

The increased ecological adaptability and environmental fitness of phage‐infected bacterial hosts present an applicable scenario to develop next‐generation bacterial inoculants involving filamentous phages infecting PGPR. These PGPR inoculums also contribute directly and indirectly in reclamation of degraded lands by facilitating plant colonization. They directly stimulate the formation and elongation of roots, improve soil nutrition status, and enhance nutrient uptake through chelated forms. Indirectly, they manage pathogens and minimize contaminant concentrations (Kaur et al. 2018). Filamentous phages must be involved in this process of outcompeting native bacteria by PGPR for colonizing the soil‐rhizosphere. The potentiation with co‐inoculation with both filamentous phages and bacteria will be the basis for successful ecorestoration (Sharma et al. 2019).

### 3.3 Phages Aid Bacterial Hosts in Adapting to Environmental Stresses

Filamentous phages have drawn the attention of microbial ecologists and biotechnologists in view of their unique morphological, physiological, and genomic features. They are single‐stranded, circular deoxyribonucleic acid viruses with an elongated spiral protein shell and no lipid membrane (King 2012). Because of their small, compact genome, it is relatively easy to engineer filamentous phages to expose different peptides or polypeptides that are capable of being displayed on the surface of such phages, rendering them useful for designing multifunctional biosensors (Petrenko 2008). Consequently, these phages have been widely researched for their conventional role in displaying specific peptides or proteins (Benhar 2001; Kehoe and Kay 2005; Willats 2002), as well as for innovative applications in developing diagnostic tools, and advancing nanobiotechnology and synthetic biology to investigate microbial secretomes (Rakonjac et al. 2011; Henry et al. 2015; Gagic et al. 2016; Székely and Breitbart 2016; Mai‐Prochnow et al. 2015).

Filamentous phages either carry genes or modulate the expression of bacterial genes, enabling their hosts to adapt to both abiotic and biotic stresses (Shapiro et al. 2016). This adaptation includes developing resistance to microbial toxins and coping with environmental stresses such as salinity, desiccation, high temperatures, and elevated contaminants (Shapiro et al. 2016; Secor et al. 2015; Yu et al. 2015).

Phages have been shown to bridge the gap from what is just observed in laboratory microbiology to a real field of application. *Pseudomonas* is, for example, able to efficiently degrade phenols in vitro but not in vivo when applied to polluted soil, water, and raw sewage (Mrozik et al. 2011).

Only partial mineralization of phenols by Pseudomonas, on which account the reliability of groundwater cleaning cannot be determined solely by this microorganism, has several reasons. The major ones are high predation or phagocytosis by phages with respect to them, brisk bacteria competition, enhanced sensitivity towards bacterial toxins, and lack of spread in Pseudomonas.

These elements reduce the efficacy and longevity of inoculums. However, there are specific phages that can address these limitations and augment the in vivo activity of bacteria. For example, bacteria infected with filamentous phages were resistant to being preyed upon by other phages as well as bacterial toxins. They were, in addition, able to move towards toxicants and were efficient at degradation (Addy et al. 2012; Chouikha et al. 2010; Yang et al. 2010).

Changes in viral community structure and patterns of phage–host interactions were examined along lateral gradients of chromium‐induced ecological stress. With increasing chromium‐driven environmental stresses, we observed a stronger positive covariation between phage and bacterial communities (represented by the higher relative abundance of lysogenic phages in viromes and a larger fraction of lysogenic bacteria being detected (Huang et al. 2021).

Moreover, viral genomic analysis showed that phages adapted to high‐level chromium stress carried an increased number of accessory metabolic genes (AMGs) implicated in aerobic chromium resistance and survival in severe environments. They also illustrate the diversity of phage–microbe interactions in challenging environments and provide new insights into the influence of phages on biogeochemical processes (Huang et al. 2021).

The relative abundance of bacterial genera, such as *Micropruina*, *Brevibacterium*, and *Bacillus*, which exhibit robust heavy metal resistance and survival under harsh conditions (Liu et al. 2018), was raised in severely Cr‐contaminated settings heavily; CRISPR‐based host linkage data disclosed a tandem rise in the prevalence of phages dependent on these hardy hosts (Shapiro et al. 2010).

This study of virome and metagenome investigated the development of phage–bacterium interactions in Cr contaminated soils. It reported that such interactions change from antagonistic (parasitic) at lower stresses to more cooperative and mutualistic as Cr pressure increases. This mutualistic interplay is dominated by lysogenic integration of temperate‐like phages. That is, lysogenic phages carry MRGs in their genomes and enhance the fitness of hosts against the toxic pressure of heavy metals by incorporation into their genomes. This insertion enhances the expression of resistance genes and mediates the replication of the resident phages. This indicates that viral populations exert a major impact on the adaptation of the prokaryotic communities to harsh environments. Knowledge on bacterial–phage interactions could also promote reclamation and restoration of heavy metal contaminated sites by modifying redox processes that render metals less toxic and mobile (Shapiro et al. 2010).

### 3.4 Phage‐Encoded AMGs as Metabolic Modulators for Advanced Bacterial Metabolic Engineering

Phage‐mediated metabolic changes in bacteria are hypothesized to markedly alter global nutrient and biogeochemical cycles (De Smet et al. 2016).

It is further proposed that phage‐mediated metabolic changes of bacteria have the potential to drive global nutrient and ecosystem cycles. De Smet et al. (2016) predicted that the number of phage‐encoded AMGs will increase exponentially in the next few years. The discovery of these “metabolic modulators” would provide critical tools for further development of what is known as “advanced metabolic engineering” in bacteria. They measured intracellular metabolite dynamics in *P. aeruginosa* cells infected with lytic bacteriophages belonging to six genera employing high‐coverage untargeted metabolomics. Our analysis of this data indicated a strong impact on host metabolism (De Smet et al. 2016).

In general, phages usually upregulate pyrimidine and nucleotide sugar metabolism. In addition, phage‐ and infection stage‐specific responses differ widely from a massive depletion of metabolites (e.g., phage YuA) to complete metabolic reprogramming (e.g., phage phiKZ). Expectedly, the most abundant metabolites homospectrally altered during infection were associated with pathways impacted by phage‐carried AMGs. Phages with AMGs capable of degrading the host genome (YuA and LUZ19). Different effects were observed at pyrimidine metabolism in phages encoding AMGs having nuclease activity (YuA, LUZ19) compared to those without genes for nucleases (phiKZ). This distinction illustrates the connectedness of a phage's unique supply of AMGs and host physiology, its impact on (De Smet et al. 2016).

## 4 Negative Effect of Bacteriophage: Phage Predation on Actinobacteria Diminishes Their Multifunctional Roles

Actinobacteria, including filamentous genera like *Streptomyces*, *Tomitella*, *Nocardiopsis*, and *Nocardia*, as well as non‐filamentous genera such as *Arthrobacter*, *Microbacterium*, *Rhodococcus*, and *Frigoribacterium*, are Gram‐positive, sporulating bacteria known for their exceptional antibiotic production (Jose et al. 2021; Montaño et al. 2022; Rahlwes et al. 2019).

The phage infection of Actinobacteria imposes both evolutionary and ecological forces on these bacteria, and offers a powerful approach to phage typing and taxonomic identification. Phages also provide additional tools to investigate actinobacterial resistance and control characteristics against the phages. Actinobacteria contribute to development, well‐being, and crop production in plants. Phage grazing may influence their ecological and metabolic roles in different habitats.

Since they are widely distributed and have the potential to synthesize a variety of antibiotics, members of *Streptomyces* play important roles in pharmaceuticals, as well as in the development of biological disease‐suppressive soils under agriculture (Ebrahimi‐Zarandi et al. 2022).

Actinobacteria, which are fundamental in agriculture and biotechnology, may be negatively affected by phage due to disturbance within their function and populations. In the pharmaceutical industry, this interference with antibiotic production can lead to financial damage. From an ecological point of view, phage infection may impact Actinobacteria functions in bioactive compounds synthesis, nutrient cycling, nitrogen assimilation, phytohormone production, or plant tissue colonizing process (Nwokolo et al. 2024).

## 5 The Potential Effects of Bacteriophages on Soil Nutrient Cycling

### 5.1 Nitrogen Metabolism and Availability

Elevated phage pressure may also cause changes in soil bacterial communities that influence microbial‐driven services. Soil bacteriophages influence nutrient cycling through stimulation of host metabolism by AMGs and lysis of bacteria involved in biogeochemical cycles (Wang et al. 2022).

AMGs participate in multiple metabolism pathways such as carbon, nitrogen, and sulfur metabolisms in addition to the photosynthesis (Bi et al. 2021; Middelboe and Brussaard 2017), also including photosynthesis (Sullivan et al. 2006).

The increase in metagenomic sequencing data over the last 10 years has led to an accumulation of several dozen AMGs from both eukaryote‐ and prokaryote‐infecting viruses. These genes are involved in a range of metabolic functions, such as phosphorus metabolism (Martiny et al. 2009) and nitrogen utilization (Sullivan et al. 2010), photosynthesis (Lindell et al. 2005; Frank et al. 2013), the pentose phosphate cycle (Thompson et al. 2011), nucleic acid biosynthesis (Miller et al. 2003), and other biochemical pathways (Sharon et al. 2011).

So far, AMGs have been found to be abundant throughout the majority of central carbon metabolic routes (Hurwitz et al. 2013). In order to more efficiently categorize the expanding numbers of AMGs, Hurwitz et al. (2015) classified annotations as belonging to one of two classes: Class I was made up of KEGG metabolic pathway‐annotated genes, while Class II contained general metabolic function (or not found in KEGG pathways), such as transport‐related functions (Hurwitz et al. 2015).

Bacteria–phage interactions also have ecological implications in the modification of nitrogen turnover. Phage pressure can shape bacterial diversity and function. It is known that phage interactions with bacteria increase soil ammonium levels. It is widely assumed that this P release is promoted via lysis of the host bacteria, from which cellular compounds such as amino acids, nucleic acids, and proteins are released into the soil, where they can be mineralized by other soil microflora (Braga et al. 2020).

Another study found that changes in microbial diversity influenced soil ammonium levels (Calderón et al. 2017). Phages release organic nitrogen from their hosts into the soil, a function they also perform in marine environments (Shelford and Suttle 2018).

Phage predation influences microbial functions in diverse ecosystems. For example, in rhizobia‐legume symbiosis, phages can diminish nodulation in susceptible rhizobia strains but enhance nodulation in legumes through phage‐resistant rhizobia strains, ultimately affecting nitrogen fixation rates in both scenarios. Experiments involving the isolation of *Enterobacter* bacteriophage and its inoculation reveal that this phage impairs soil nitrogen fixation by destroying nitrogen‐fixing bacteria and disrupts the diversity and composition of soil bacterial communities (Wang et al. 2022).

Calderón et al. (2017) and Braga et al. (2020) found that higher NH_4_^+^ levels could stem from phage‐induced host cell lysis, which releases inorganic nitrogen that is then mineralized. This aligns with similar observations of organic nitrogen being released during viral lysis in marine environments.

### 5.2 The Impact of Viruses on Carbon Cycling and Soil Organic Matter Mineralization

One of the major concerns for the environment today is climate shift, with one of its significant impacts being the increased concentration of atmospheric carbon dioxide (CO_2_) (Conant et al. 2011). Soil CO_2_ emissions arise from organic matter breakdown, microbial processes, and root respiration, with soil moisture and temperature playing key roles in these activities (Pumpanen et al. 2015; Zhou et al. 2016).

It was postulated that the increased abundance of T4‐like phages could, in turn, lead to an increase in bacterial mortality resulting from decreased abundance of T4‐susceptible bacteria and therefore suppress SOC mineralization. Soil CO_2_ emission increased with bacterial abundance and decreased when T4‐like phages were more abundant. The random forest model also revealed that the abundance of T4‐like phages and their ratio to bacterial abundance are better quantitative predictors for SOC mineralization (measured as CO_2_ efflux) in comparison with only purely using bacterial abundance. Experimental evidence sheds light on the implications of phages in SOM cycling. They may retard the decomposition of soil organic carbon and yet accelerate bacterial turnover (Wei et al. 2021).

Using the dbCAN meta server (http://bcb.unl.edu/dbCAN2/index.php), accessed May 20, 2022, 100 auxiliary carbohydrate‐active enzyme (CAZyme) genes were identified in the viruses, potentially aiding host bacteria in carbon metabolism. The identified CAZyme genes belong to five classes: Auxiliary Activities (Yan et al. 2023), Carbohydrate Esterases (Sullivan et al. 2010), Glycoside Hydrolases, Glycosyl‐Transferases, and Polysaccharide Lyases, along with one module: Carbohydrate‐Binding Modules (Yan et al. 2023).

### 5.3 Carbone Degradation: Pollutant Degradation

Previous research on soil viruses has shown that they infect bacteria with crucial biogeochemical functions, such as *Acidobacteria*, *Verrucomicrobia*, and *Deltaproteobacteria*. Given that these viruses encode glycoside hydrolase genes involved in complex carbon degradation, they may significantly influence soil carbon cycles (Emerson et al. 2018).

Zheng et al. (2022) used metagenomics to reveal that organochlorine pesticide‐contaminated soil had lower bacterial diversity but a higher diversity of viruses. These viruses carried a greater proportion of AMGs involved in pesticide breakdown and metabolism, aiding bacteria in coping with pesticide stress (Zheng et al. 2022).

Additionally, they identified numerous biodegradation genes within viral metagenomes, noting a significant increase in diversity and relative abundance of AMGs correlated with the severity of pesticide contamination. They also observed that viral‐encoded L‐2‐haloacid dehalogenase genes (L‐DEX) facilitated the degradation of L‐2‐haloacid pesticide precursors, enhancing bacterial growth at sub‐inhibitory pesticide concentrations (Zheng et al. 2022).

Microbiomes in organic fertilizers significantly enhance soil ecology by improving soil structure, increasing crop production, and aiding in pollutant degradation. Another study identified phage AMGs linked to metabolic processes and pesticide degradation present throughout organic fertilizers (Chao et al. 2023).

Chao et al. (2023) demonstrated that vermicomposting enhanced organic fertilizers by increasing phage‐host pairings and diversifying phage AMGs compared to non‐composted fertilizers. The study also found that vermicompost phages efficiently aided in breaking down the pesticide precursor p‐nitrochlorobenzene in soil. These results emphasize the importance of these phages in driving biogeochemical processes and facilitating the degradation of pesticide‐associated substances in polluted soils (Chao et al. 2023).

### 5.4 Phosphorus (P) Metabolism

Phosphorus (P) deficiency presents a significant challenge in agricultural ecosystems (Kirkby and Johnston 2008). Earlier studies showed that viral communities exhibit greater adaptability to shifts in soil nutrient levels compared to prokaryotic communities. In particular, the relative abundance of *Caudoviricetes* markedly decreased as soil phosphorus availability and total nitrogen levels increased (Huang et al. 2024).

Furthermore, they discovered several potential AMGs involved in phosphorus and nitrogen cycles, such as *phoA, phoH, phoB*, and *gdh*, with *phoB* and *gdh* being identified for the first time as AMGs encoded by soil viruses (Huang et al. 2024).

The diversity and distribution of viral *phoH* are mainly influenced by the environment. The phosphorus metabolism genes of these viruses may contribute to the nucleotide supply for viral reproduction in bacterial hosts as well, and consequently affect phosphorus pathways in soil environments. Interestingly, half of the key proteins for phosphorus use and nucleotide synthesis (*dUTPase*, *MazG*, *PhoH*, *ThyX*, and *RNR*) were mainly enriched in agri‐soils (Han et al. 2022).

In acid mine drainage environments, genes associated with phosphorus (P) metabolism are believed to help organisms cope with phosphorus scarcity (Chen et al. 2015; Liang et al. 2020). Seventy‐five viral genes were detected and classified as phosphate starvation‐inducible proteins (*phoH*) (Hsieh and Wanner 2010).

The universal abundance of *phoH* genes increased significantly with a decrease in the concentrations of TP and AP in the sediments, suggesting that these viral *phoH* genes could be induced by phosphorus deprivation in AMD sediments. As the AMD and similar ecosystems are generally low nutrient, the identified phosphorus metabolism‐related genes may allow viruses to increase or sustain host phosphorus uptake that would represent an important adaptation strategy in this extreme environment (Gao et al. 2022).

Bacteriophages also affect environmental processes like Enhanced Biological Phosphorus Removal (EBPR) by disrupting the microbial community and altering the effectiveness of phosphorus removal in activated sludge systems (Barr et al. 2010).

EBPR is a prevalent wastewater treatment technique designed to extract both phosphorus and carbon. This process involves the uptake and storage of phosphate within specialized microorganisms known as polyphosphate‐accumulating organisms (PAOs) (Liu et al. 2001).

Key PAOs involved in EBPR include Rhodocyclus‐related *Betaproteobacteria* and *Candidatus Accumulibacter phosphatis* (*Accumulibacter*) (He et al. 2007; Crocetti et al. 2000). Bacteriophage infections targeting dominant PAO populations can lead to diminished performance in EBPR. In activated sludge wastewater treatment plants, bacteriophage concentrations range from 4.2 × 10^7^ to 3.0 × 10^9^ per milliliter (Otawa et al. 2007).

### 5.5 Sulfur Metabolism: Phage Treatment of Sulfate‐Reducing Bacteria (SRB) as a Potential Strategy for Biocorrosion Control

In wetland ecosystems, bacteriophages can target sulfate‐reducing and methanogenic bacteria, conceivably inhibiting sulfate reduction (and related carbon decomposition) as well as methane production (Dalcin Martins et al. 2018).

Dissimilatory Sulfur Metabolism represents the primary pathway of sulfur metabolism on Earth, encompassing two fundamental biochemical processes: the reduction of sulfate to sulfide and the subsequent oxidation of sulfide or thiosulfate to sulfate. These reactions are critical for the global sulfur cycle and play a key role in various ecological and geological processes. The families Siphoviridae, Myoviridae, and Podoviridae include viruses known as “sulfur phages,” which interact with Microorganisms engaged in sulfur processes. These phages encode AMGs essential for sulfur metabolism (Kieft et al. 2021).

SRB can pose considerable challenges to both industrial processes and the health of humans and animals. Using phage therapy to target and inhibit SRB presents a promising approach to mitigating these adverse effects (Kushkevych et al. 2021).

The anaerobic respiration of SRB produces hydrogen sulfide (H_2_S), which can lead to numerous issues. One notable problem is the corrosion of steel parts in equipment used for oil extraction, transport, and processing due to H_2_S release (Tang et al. 2010).

The oil industry is one of the most critical areas where the efficiency of phage therapy in controlling SRB can be ineffective. The use of chemical disinfectants is varied to control biocorrosion, which causes considerable damage in oil processing plants. Oil pipelines are treated with anti‐microbial agents to kill some of these pathogenic organisms. Nevertheless, these agents are usually ineffective against bacteria in biofilms, as the extracellular matrix produced by cells of the organisms normally serves as a resistant protector against damage (Xu et al. 2018). Alternatively, bacteriophages could enzymatically break down the polysaccharide structure, resulting in cell lysis.

In 2009, the United States awarded a patent to control biocorrosion with bacteriophages (Xu et al. 2017). The method is to detect and separate the microorganisms that lead to corrosion in pipes and equipment, before concocting an individual cocktail of bacteriophages aimed at inhibiting their growth. Phages from previously established libraries or newly isolated are adapted by mutagenesis or genetic engineering. These are then sent to the site through a long, cylindrical device called a “pig,” which pumps infected fluid or gel into pipelines. It also addresses “acid” oil issues by inoculating SRB that produce H_2_S, and a portion of the composition is designed for bacteria in water to help keep up pressure and well shots. It solubilizes two bacterial groups, which are known to be involved in oil degradation (Summer 2009).

The primary SRB phages are from the Siphoviridae and Myoviridae families (Gong 2010). Specifically, the lysogenic bacteriophage from the Siphoviridae family targets and lyses salt‐dependent SRB, such as *Desulfovibrio salexigens*.

### 5.6 Enhancing Plant Growth With Magnesium and Manganese Nanoparticles Synthesized by Bacteriophages

Magnesium (Mg), crucial for processes like photo‐assimilation and photophosphorylation, is absorbed by plants in nanoparticle or ionic forms. Tamil Elakkiya et al. (2020), reported MgONPs can increase chlorophyll content by six times.

Nano‐fertilizers composed of different metal oxides present benefits like reducing nutrient loss from runoff or evaporation, enhancing absorption efficiency, and offering greater biodegradability than standard fertilizers (Huang et al. 2018; Salas‐Leiva et al. 2021).

Manganese is a crucial micronutrient for plant growth, playing a vital role in sustaining metabolic functions. It is recognized as a key cofactor in the oxygen‐evolving complex of photosynthesis, where it significantly enhances the water‐splitting reaction in photosystem II (PSII) (Broadley 2012).

Bacterial leaf blight (BLB), caused by *Xanthomonas oryzae* pv. *oryzae* (Xoo), is a significant disease affecting rice plants with substantial economic impact (Yasmin et al. 2017).

In a recent study, MgO and MnO_2_ nanoparticles (MgONPs and MnO_2_NPs) were synthesized and characterized using the lysate of *X. oryzae* bacteriophage X3, a member of the Myoviridae family (Ogunyemi et al. 2019).

The study explored how nanoparticles mediated by bacteriophages affect plant photosynthesis, influence genes related to chlorophyll production, and provide protection against BLB (Ogunyemi et al. 2023). They observed a notably higher expression of chlorophyll synthesis and photosystem structure genes at all concentrations of MgONPs and MnO_2_NPs compared to the control. Additionally, the nanoparticles significantly enhanced chlorophyll fluorescence parameters, protected plants from bacterial infection by inhibiting rice BLB, and did not exhibit toxic effects on Arabidopsis (Ogunyemi et al. 2023).

## 6 Bacteriophage‐Based Biocontrol Technology to Enhance the Efficiency of Wastewater Treatment

Because treated wastewater is increasingly reused for agricultural irrigation, the efficiency of wastewater treatment directly affects soil quality and food safety. The infiltration of contaminated water and wastewater into agricultural soil can compromise soil quality and impact the safety of agricultural products. Therefore, it is crucial to examine both the chemical and microbial contamination present in these waters and assess the effectiveness of wastewater treatment systems (Bayabil et al. 2022; Xu et al. 2010).

A portion of the nutrients used in agriculture and urban landscapes eventually washes away into runoff, contributing to the pollution of streams, rivers, lakes, groundwater, and other water bodies (Bayabil et al. 2022).

Although long‐term irrigation with effluent offers the benefits of organic matter and nutrient enrichment to agriculture, it carries a risk. Heavy metals and other trace contaminants that accumulate in topsoil may deteriorate soil and groundwater quality, which will threaten the sustainability of land effluent disposal (Xu et al. 2010).

Wastewater reuse for irrigation – Treated wastewater is used in many countries worldwide to irrigate crops as an approach to overcome water scarcity. The long‐term sustainability of the use of non‐conventional irrigation water requires periodic evaluation of soil quality (Ibrahimi et al. 2022).

Biological wastewater treatment (BWT) has been recognized as an environmentally friendly and cost‐effective technology for removing pollutants from industrial and municipal wastewater, involving multiple bacterial communities that efficiently remove organic material, nitrogen, and phosphorus. Active removal of pollutants is the outcome of these cooperative interactions between bacterial groups (Liu et al. 2021). Aerobic BWT could also benefit from bacteriophages, which have the potential to enhance the treatment process by targeting specific bacterial species, leading to improved microbiota dynamics and overall effectiveness in COD removal (Reisoglu and Aydin 2023).

The activated sludge process (ASP) is one common form of aerobic treatment method in the treatment of wastewater, which can decrease organic matter consumption by microorganisms (Rustum 2009). Foaming is a significant problem in the ASP due to filamentous organisms and by gas bubble release. Increased filamentous bacteria in the sludge can result in brown, viscous foam on the surface of activated‐sludge basins and secondary clarifiers. Such foam can cause a number of operating problems (Pal et al. 2014).

Applying a range of lytic phages as a sustainable solution for managing filamentous bacteria in ASP may effectively diminish foam formation in the treatment plant (Weinbauer 2004; Withey et al. 2005) and deliver a long‐term, cost‐efficient method for managing potentially harmful bacteria (e.g., *E. coli* and *Salmonella*) (Withey et al. 2005). Additionally, *Salmonella* phages were detected at very high levels (10^2^–10^5^ PFU/mL) in all examined wastewater types.

Bacteriophages are abundant in places where bacteria exist. In activated sludge systems, the phage number ranged from 10^8^ to 10^9^ PFU/mL (Otawa et al. 2007; Brown et al. 2015).

One new strategy used to solve the pollution in wastewater is the use of polyvalent phages to selectively prevent harmful bacteria. The practice of in situ addition of polyvalent phage mixtures is an emerging, fast, and economical means for reducing pathogen hazards and improving water quality in wastewater treatment.

The polyvalent phage cocktails presented particle morphologies characteristic of *Siphovirus*, *Myovirus*, and *Podovirus* isolates and displayed broad lytic activity toward multiple multidrug‐resistant bacteria, including *E. coli*, *Citrobacter freundii*, *Proteus vulgaris*, *Salmonella* species, *P. aeruginosa*, *Pseudomonas fluorescens*, and *Enterococcus faecalis* (Azzam et al. 2024).

Anaerobic digestion of secondary waste‐activated sludge in the wastewater treatment process not only eliminates sludge but also produces methane, a renewable bioenergy resource. In anaerobic digestion, hydrolysis of secondary sludge is one of the initial stages and a rate‐limiting step.

Kim et al. (2022) demonstrated that applying bacteriophage lysozymes notably enhances sludge reduction. Their study used lysozymes from bacteriophage species T4, T7, and λ in batch tests to accelerate the hydrolysis of secondary sludge (Kim et al. 2022).

## 7 Bacteriophage Dispersal in the Rhizosphere Through Animal Taxa

### 7.1 Animal‐Mediated Bacteriophage Dispersal Mechanisms in the Rhizosphere

The process of sustaining infection cycles for bacteriophages is highly dependent upon the efficient transfer of the bacteriophages between their hosts. Bacteriophages have no capability for self‐transfer (van Sluijs et al. 2025). Animals do not interact directly with bacteriophages to their advantage; instead, they can significantly alter the rhizosphere environment by amplifying, mobilizing, and distributing both bacterial populations and phages that infect them. Herein, three main animal‐mediated bacteriophage dispersal mechanisms in the rhizosphere are suggested. First, dispersal occurs through fecal excretion. Animals consume soil and organic material components found in the rhizosphere, which is rich in bacteria and phages. Second, phages can be transported by animal vectors. Phages can adhere to exterior surfaces of animals, such as their legs, hairs, fur, and cuticles. As animals move through the rhizosphere, they carry these phages to distant locations. Third, phage transport occurs due to physical mixing or disturbance of the soil caused by animal movements and burrowing behaviors. A wide variety of terrestrial invertebrates and vertebrate animals, such as earthworms, nematodes, ants, centipedes, millipedes, rodents, moles, and other burrowing species, mix and alter soil structure by creating tunnels, digging burrows, or plowing it. These activities help move phages within the rhizosphere. In addition, this physical mixing can change the oxygen concentrations in the rhizosphere, which, in turn, influences the structure of bacterial communities between aerobic and anaerobic organisms. Consequently, these changes also affect the composition of the resident phage community.

### 7.2 Ecological Categorization of Animals Based on Their Role in Bacteriophage Transportation Within the Rhizosphere: From Local to Long‐Distance Dissemination

**Figure 1:**
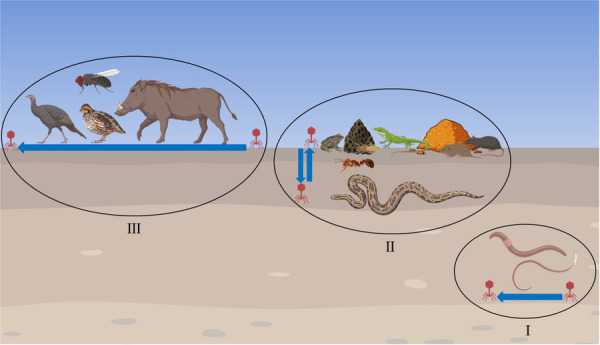
Animal‐mediated bacteriophage dispersal mechanisms in the rhizosphere: I: The soil‐inhabiting animals live almost entirely under the ground, inside or beneath the rhizosphere. Examples include earthworms and nematodes; II: The burrowing animal shuttles between their burrows and the soil's surface; III: Surface dwellers live mostly on the soil surface and can move phages across great distances. The figure was created by Biorender.

According to the ecological habitat and dispersal ability, animals can be classified into three categories (Figure 1, Table 1) (Zhong et al. 2020; Yuan et al. 2020).

**Table 1:** Inventory of isolated bacteriophages, their animal vectors, and host bacteria.

Animal taxa	Animal species	Ecological category of animals	Bacteriophage	Host bacteria	Studied animal samples	References
Earthworm	*Eisenia andrei*	Soil dweller	EF1	*Citrobacter freundii* *Citrobacter braakii* *Citrobacter murliniae*	Casting	Jung et al. (2016)
Earthworm	*Eisenia fetida*	Soil dweller	Wip1, Wip4	*Bacillus anthracis*, *Bacillus cereus*	Intestine	Schuch et al. (2010)
Earthworm	*Alma emini*	Soil dweller	*Cystoviridae, Podoviridae, Myoviridae*	Not mentioned	Digestive tract and soil	Nana et al. (2015)
Earthworm	*Metaphire guillelmi*	Soil dweller	*Mimivirus*	*Enhydrobacter* sp.	Intestine	Xia et al. (2023)
			*Myoviridae*	*Enterobacter cloacae*, *Aeromonas caviae*, *Clostridium botulinum*, *Paracoccus denitrificans*, *Variovorax paradoxus*, *Vibrio cholera*, *Lactobacillus* sp.		
			*Podoviridae*	*Massilia* sp.		
			*Siphoviridae*	*Avibacterium*, *Paragallinarum*, *Gammaproteobacteria bacterium*, *Leclercia adecarboxylata*, *Pantoea agglomerans*, *Pararheinheimera mesophila*, *Streptomyces* sp.		
Earthworm	*E. fetida*	Soil dweller	Wip1, Wip2, Wip4, Wip5	*B. anthracis*	Earthworm gut, or earthworm gut bacteria	Schuch and Fischetti (2009)
Nematods	*Caenorhabditis elegans*	Soil dweller	Min1	*Microbacterium nematophilum*	Not mentioned	Akimkina et al. (2007)
Insect (ant)	*Linepithema humile*	Burrow‐nesting	PS‐1	*Pseudomonas*	Not mentioned	Baty et al. (2020); Lester et al. (2019)
			SJ46	*Salmonella*		
			SfIV	*Shigella*		
Insect (aphid)	*Acyrthosiphon pisum*	Soil surface‐dweller	APSE	*Hamiltonella defensa*	Endosymbiont	Weldon et al. (2013); Rouïl et al. (2020)
Insect (termite)	*Coptotermes formosanus*	Burrow‐nesting	AR9	*Bacillus*	Gut	Tikhe and Husseneder (2018)
			ProJPt‐Bp1	*Azobacteroides*		
			c‐st	*Clostridium*		
			phiR1‐37	*Yersinia*		
			SP‐15	*Bacillus*		
			P2559Y	*Croceibacter*		
			PAU	*Sphingomonas*		
			vB_BanS‐Tsamsa	*Bacillus*		
			PaV‐LD	*Planktothrix*		
			Sundance	*Brevibacillus*		
			BM5	*Bacillus*		
			vB_BhaS‐171	*Bacillus*		
			phiCD211	*Clostridium*		
			OBP	*Pseudomonas*		
			BP63	*Salmonella*		
			0305phi8‐36	*Bacillus*		
			P2559S	*Croceibacter*		
Insect (termite)	*C. formosanus* and *Coptotermes gestroi*	Burrow‐nesting	TG‐crAlp‐04, TG‐crAlp‐06	*Azobacteroides pseudotrichonymphae*	Protozoa in the termite gut	Chen et al. (2022)
Insect (fly)	*Hermetia illucens* larvae	Soil surface‐dweller	PHB10	*Escherichia*	Gut	Chen, Li, et al. (2019b)
Insect (Scarabid beetles)	*Protaetia brevitarsis* larvae	Soil dweller	SV1, Phic31, TG1, phiBT1, phiSASD1	*Streptomyces*	Gut	Min et al. (2025)
			S1	*Stenotrophomonas*		
			bIL312, bIL310, bIL309	*Lactococcus*		
			E2	*Geobacillus*		
			HK639, P1	*Escherichia*		
			phIS3501, phBC6A52, phBC6A51	*Bacillus*		
			Papyrus, PBI1, Wanda, U2, Bethlehem, PattyP, LittleCherry, CASbig, Fishburne, Che9c, Che8, WIVsmall, Velveteen, Hamulus, Bobi, Brujita, Bxz1	*Mycobacterium*		
			phi_OH2	*Thermus*		
			phiAsp2	*Actinoplane*		
			CP2	*Xanthomonas citri*		
			BcepC6B	*Burkholderia*		
Myriapoda	*Epibolus pulchripes*	Burrow‐nesting	Caudoviricetes (24 phages)	Bacillota, Pseudomonadota, Desulfobacterota, Actinomycetota, and Spirochaetota	Gut	Nweze et al. (2024)
Myriapoda	*Glomeris connexa*	Burrow‐nesting	Caudoviricetes (17 phages)	Pseudomonadota and Bacillota	Gut	Nweze et al. (2024)
Woodlice	*Armadillidium vulgare*	Burrow‐nesting	WO	*Wolbachia*	Not mentioned	Martin et al. (2010)
Mammals	Tree shrew (*Tupaia belangeri*)	Burrow‐nesting	phiEaH2	*Erwinia*	Feces	Chen, Gu, et al. (2019a)
			phiEF24C	*Entrococcus*		
			SPN3US	*Salmonella*		
			ECML‐117	*Escherichia*		
			Bcp1	*Bacillus*		
Birds	Wild pigeon (*Columba livia*)	Soil surface‐dweller	Ec_MI‐02	*Escherichia coli*	Feces	Sultan‐Alolama et al. (2023)
Birds	Quail	Soil surface‐dweller	IME‐EFm5, IME‐EFm1, EF1, EFP01, EFDG1, EFLK1, ECP3, IME_EF3	*Enterococcus*	Intestine	Xiong et al. (2024)
			XacN1	*Xanthomonas*		
			MG‐B1, *Bacillus* phage Silence	*Bacillus*		
			Dp‐1	*Streptococcus*		
			EcoDS1	*Enterobacteria*		
			P118	*Lactococcus*		
			LMTA‐148	*Listeria*		
			ST2, ST31	*Escherichia*		
Birds	Pigeon, goose, and chicken	Soil surface‐dweller	vB_EcoM_P3322	*E. coli*	Diarrhea	Tong et al. (2025)
Reptiles	Savannah monitor (*Varanus exanthematicus*)	Burrow‐nesting	pSal‐SNUABM‐04	*Salmonella*	Skins and cloaca	Kwon et al. (2020)

Group 1 – The soil‐inhabiting animals live almost entirely under the ground, inside or beneath the rhizosphere. Examples include earthworms and nematodes. These animals efficiently turn over small volumes of soil through their feces and soil movement (Witmer et al. 2012).

Group 2 – The burrowing animals shuttle between their burrows and the soil's surface. These include animals like rodents, meerkats, snakes, lizards, toads, ants, beetles, and myriapods. Acting as bioturbators, they move phages while digging, excreting waste, and moving around (Wang and Hou 2021; Lu et al. 2025).

Group 3 – Surface dwellers live mostly on the soil surface and can move phages across great distances. The wild boar, bear, ground‐feeding birds, and even flies and aphids, which have little contact with the soil, belong to this group. They can transfer their phages to other areas via their feces (He et al. 2022; Raghwani et al. 2023).

### 7.3 The Role of Invertebrates in Phage Dispersal in the Rhizosphere

Invertebrates have microbes present in the gut, salivary glands, hemolymph, and reproductive system. Where there are bacteria, there are phages (Don et al. 2019; Tomita and Hiura 2022). Below is a summary of different invertebrate groups and how they help in phage dispersal.

#### 7.3.1 Earthworms

Earthworms significantly modify the soil habitat by burrowing, feeding, and casting. Endogeic or epigeic, even anecic earthworms will mix soil horizons and change their makeup. This increases aeration, improves water management in the soil, and increases nutrient availability. Furthermore, they generate hotspots of microbial activity in the gut itself and on the resulting casts (Blouin et al. 2013; Bertrand et al. 2015; Fattore et al. 2020; Lejoly et al. 2024). Earthworms greatly affect the soil through activities such as burrowing, feeding, and defecation. An individual earthworm displaces more than 0.5 kg of soil per year. With thousands of earthworms per acre, they displace 10 to 40 tons of soil/acre/year, creating 0.1 to 0.4 cm of soil layer every year (Blouin et al. 2013; Kutschera and Elliott 2010). The gut of earthworms creates a special environment where large numbers of bacteria flourish, hence producing lots of phages (Wang et al. 2024). They carry phages in their droppings as well as in their bodies, and they are predated by birds, mammals, and reptiles who can carry phages elsewhere (Řezáč et al. 2018; Chitty 2022; Jung et al. 2016). Several bacteriophages have been isolated from earthworm guts or casts (Table 1).

#### 7.3.2 Nematodes

One group of organisms that have been found to be abundant soil inhabitants is nematodes, with densities of up to 10 million per square meter having been reported (van Sluijs et al. 2025). Experiments have demonstrated that *Caenorhabditis elegans* and *C. remanei* act as vectors for phage transmission in soil (van Sluijs et al. 2025; Dennehy et al. 2006).

#### 7.3.3 Arthropods

Arthropods (insects, arachnids, myriapods) play an important role in improving the motility of bacteriophages by means of consumption, defecation, and movement (Gange 2000; Potapov et al. 2022). They are considered bio‐turbators that incorporate the upper layers of soil into the lower ones by digging (Jouquet et al. 2006).

Ants and termites construct large colonies inside the rhizosphere, while spiders, scorpions, centipedes, and beetles destroy the soil structure during the construction of burrows. Some arthropods (oribatid mites, beetle larvae) are soil‐inhabiting and disperse phages over small distances. Some arthropods (ants, wasps, termites, centipedes, and arac) are burrow‐habitating and accomplish moderate dispersion of phages. Surface arthropods (grasshoppers, crickets, various beetles) disperse phages over long distances by means of excreta or by attaching to their cuticle (Barczyk et al. 2024; Avila‐Arias et al. 2022; Řezáč et al. 2018; Chitty 2022). Table 1 provides an inventory of phages isolated from arthropod vectors. In addition, insects like aphids and flies can also be responsible for the long‐distance transport of phages, despite having limited contact with the rhizosphere (Table 1).

### 7.4 The Role of Vertebrates in Phage Dispersal in the Rhizosphere

Vertebrates travel over longer geographical ranges and help in spreading phages via defecation, transport, and soil disruption (Table 2).

**Table 2:** Phage dispersal mechanisms through vertebrate activities.

Dispersal mechanism	Example vertebrate taxa	Effect on phages
Defecation	**Many terrestrial vertebrates.** Ruminants, elephants, rodents songbirds (e.g., robins, starlings), landfowl, ostriches monitor and spiny‐tailed lizards, tortoises	**Creates localized amplification hotspots.** Defecation creates localized, nutrient‐rich hotspots that trigger massive bacterial growth and consequent phage amplification. Provides long‐range dispersal.
Burrowing	**Primary or secondary burrowers.** Rodents, hedgehogs, shrews, moles, wombats, badgers, meerkats burrowing Owl iguanas, tortoises, crocodiles, snakes, monitors, geckos, toads	**Large‐scale mixing and vertical transport.** Redistributes phages through the rhizosphere and creates new microbial habitats on burrow walls.
Digging/foraging/plowing	**Ground‐foragers.** Wild boars, bears, armadillos, badgers, and anteaters landfowls (e.g., chickens, turkeys, Megapodes) turtles, skinks	**Localized mixing and dispersal.** Physically disturbs the soil surface, redistributing phages and altering the soil environment for their bacterial hosts.
External transport	**Many terrestrial vertebrates.** Burrow‐nesting mammals, birds, and reptiles. Migratory birds and mammals.	**Ultra‐long‐distance dispersal.** Phages adhere to fur, scale, hair, and feet, allowing them to be vectored across the rhizosphere, connecting distant microbial communities.

#### 7.4.1 Mammals

Burrow nesting mammals (mole, hedgehog, ground squirrel, vole, meerkat) spread phages through their extensive tunnel networks (Witmer et al. 2012; Wang and Hou 2021; Pinkert et al. 2025). These mammals can be classified as: (a) primary burrowers that dig their own shelters, or (b) secondary burrowers that inhabit shelters constructed by others (Table 2).

Mammals, especially rodents, play a key role; Yersinia pestis phages have been obtained from squirrels and *Rattus norvegicus* (Zhong et al. 2020; Yuan et al. 2020); caudoviricetes and Microviridae phages have been detected in voles and pikas of the Qinghai‐Tibet Plateau (Lu et al. 2025; He et al. 2022), and multiple families of phages were obtained from *Apodemus* and *Myodes* genus metagenomes (Raghwani et al. 2023). Mammals that disturb the surface layer (bears, wild boar, badgers, armadillos) dig up the soil when they forage for food, promoting phage migration in the topsoil layer of the rhizosphere (Don et al. 2019; Tomita and Hiura 2022; Jones et al. 2025; Carpio et al. 2022).

#### 7.4.2 Birds

Many bird species are associated with the rhizosphere due to their nesting, feeding, and burrowing behavior. Birds that feed on the ground (quails, pheasants, turkeys, megapodes) dig into the soil surface, thus stirring the phages (Kofron 2024; Holt et al. 2023; Saibi et al. 2022).

Burrowing nesting animals (bee‐eaters, sand martins, burrowing owls) dig or make use of burrows, thus contributing to the distribution of phages (McGowan et al. 2018; De Marchi et al. 2017). New bacteriophages have been identified in the feces and intestinal content of birds (Table 1) (Xiong et al. 2024).

#### 7.4.3 Reptiles and Amphibians

Phages carried by reptiles include those that are transported in their feces, externally, and by disturbing the soil. The majority of reptiles are burrow‐nesters: tortoises dig holes meters long; iguana females create burrows of about 2 m in length where they lay eggs; mugger crocodiles dig burrows of 10 m or more (Moore et al. 2018; Harris et al. 2015; Du et al. 2023; Whitaker et al. 2007; Mobaraki et al. 2013). Snakes and pythons make use of existing burrows (Hollandt et al. 2021; Dorfman et al. 2023). Burrowing toads, among other amphibians, may also carry phages via soil disturbance and digestion of prey (Oromí et al. 2010; Silva et al. 2016). Nevertheless, research into bacteriophages in reptiles and amphibians still lacks (Lu et al. 2022; Orton et al. 2020).

## 8 Conclusion

Bacteriophages are plentiful and highly active inhabitants of soil communities, impacting soil processes, bacterial populations, and plant growth. The bacteriophages have important roles in soil ecosystems, where they lower the pathogenicity of phytopathogens, improve bacterial inocula, help hosts adapt to stressful environmental conditions, and regulate metabolism using AMGs. But at the same time, these positive effects are accompanied by adverse influences such as interference with the activity of symbiotic nitrogen fixers and inhibition of the activities of beneficial bacteria.

Another dimension of viral spread that receives comparatively little attention but is just as important is phage dispersal. The phages lack any means of active locomotion and rely exclusively on animal vectors for their dispersal from centimeters in range (invertebrate soil dwellers) up to kilometers (migratory birds and mammals) via fecal discharge, external transport, and soil manipulation. The ecological classification described above (soil‐dwellers, burrow‐nesters, surface‐dwellers) can serve as a basis for predicting phage dispersal potential depending on the animals' behaviors and habitats.

Further research is recommended to focus on the following directions. First of all, quantitative analysis of phage dispersal distance and velocity by various animal groups is highly desirable. Second, the importance of phages with AMGs in the animal microbiome is a largely underexplored field. Lastly, coupling phage dispersal models with soil management approaches may help develop biological control methods (i.e., utilizing earthworms or ants for phage transport to targeted bacteria). We also suggest that future reviews contain schematic figures for each main chapter to present complicated data in an illustrative manner, as recommended by the reviewer.

## Author Contributions

**Majid Komijani:** supervision, conceptualization, investigation, methodology, writing – review and editing, validation, project administration. **Hassan Maddahi:** conceptualization, investigation, methodology, writing original draft, writing – review and editing, validation. **Marzieh Rezaei:** validation, methodology. **Mohammad Hussein Abnosi:** writing – review and editing, methodology. **Abdullah Khalaf Ahmed:** writing – original draft, investigation, visualizing.

## Funding

The authors have nothing to report.

## Ethics Statement

The authors have nothing to report.

## Conflicts of Interest

The authors declare no conflicts of interest.

## Declaration of Generative AI in Scientific Writing

AI was partially used for grammar checking.

## Data Availability

The data that supports the findings of this study are available within this article.
